# Mirrors Improve Rabbit Natural Behavior in a Free-Range Breeding System

**DOI:** 10.3390/ani9080533

**Published:** 2019-08-06

**Authors:** Vincenzo Mastellone, Fulvia Bovera, Nadia Musco, Valentina Panettieri, Giovanni Piccolo, Anna Scandurra, Carmelo Di Meo, Youssef A. Attia, Pietro Lombardi

**Affiliations:** 1 Department of Veterinary Medicine and Animal Production, University of Napoli Federico II, Via F. Delpino 1, 80137 Naples, Italy; 2 Department of Biology, University of Napoli Federico II, Via Cintia, 80126 Naples, Italy; 3 Arid Land Agriculture Department, Faculty of Meteorology, Environment and Arid Land Agriculture, King Abdulaziz University, Jeddah 21589, Saudi Arabia

**Keywords:** environmental enrichment, stress markers, animals’ welfare, behavioral repertoire

## Abstract

**Simple Summary:**

The study showed that the use of mirrors may represent a low-cost useful tool to stimulate the expression of natural behaviors when rabbits are bred in small groups in a free-range system. The use of mirrors appeared to simulate the condition in which rabbits were allowed to have visual contacts with the other groups, improving important natural behaviors (olfactory investigation, gnawing, alertness, allo-grooming, stretching, locomotion). This is important in the view of adopting welfare-friendly techniques in rearing of farm animals, since consumers demand for high quality animal products is changing in a way that includes in the concept of home-made products also alternative rearing systems complying with the conditions of welfare.

**Abstract:**

The aim of this research was to evaluate the possible usefulness of mirrors in improving rabbit behavior in a free-range breeding system. Three groups (each consisting of nine replicates of three animals) were compared: isolated, isolated with mirrors and separated by a wire mesh (possible visual and olfactory contacts). Rabbits allowed to have a visual and olfactory contact showed a significantly higher expression of important natural behaviors (olfactory investigation, gnawing, alertness, stretching, locomotion) compared to the isolated rabbits (with or without mirrors); while rabbits in the mirror group showed higher allo-grooming activity than those isolated and no different locomotion activity than those separated by wire mesh. Thus, mirrors seemed to be able to modify the behavioral repertoire of isolated rabbits by acting on social perception in rabbits reared in small groups in a free-range system. Nevertheless, despite it being advisable to use a combination of different indicators in order to assess the stress level of an animal, the accuracy of serum cortisol, as well as of some secondary stress markers assay, appeared to be limited in this type of breeding.

## 1. Introduction

Public opinion strongly asks for adopting welfare-friendly techniques in rearing of farm animals. Also, consumer demand for high quality animal products is increasing [1], in particular, home-made products or meat from alternative rearing systems complying with the conditions of welfare are more required in recent years. Recently, it has been proposed to change the housing system in pairs within wire-net cages (2 rabbits/cage) or small groups (4–8 rabbits/cage), which is currently implemented for rabbits in the European countries, to adopt alternative pen housing in order to ameliorate animal behavior in respect of growth performance and meat traits [2]. Understanding of rabbit behavior is essential to increase the knowledge concerning species requirements and to ensure adaptation to intensive rearing systems. Exhibition of normal behavior is among five basic freedoms forming animal welfare specified by Farm Animal Welfare Council [3], i.e., freedom from hunger and thirst, from inadequate environment, from pain, injuries and distress, from “fear” and from the impossibility to express the “normal” behavioral repertoire. Keeping animals in an improper way leads to abnormal behavior, known as stereotypies, invariant behavior with no obvious goal of function that negatively affects animals and poor psychological well-being in these animals [4]. The understanding of specific behaviors in wild animals is the first step to investigate on rearing strategies that can be respectful of animal welfare. Wild rabbits spend most of their resting time in groups and in close contact, demonstrating a complex social activity that cannot be duplicated under some commercial rearing conditions, thus affecting the expression of different behaviors and the quality of social interactions [5]. Numerous studies have been conducted on the effects of housing system and environmental enrichments (e.g., plastic platform, hiding box, gnawing stick, mirrors) to alleviate this problem, especially with regard to productive performance and the welfare of growing rabbits [6,7,8,9]. In particular, some authors compared whether the presence of mirrors in cages or pens influenced rabbit behavior [7,10,11]. The limitations of the above-mentioned researches were that none of them directly measured stress, fear or anxiety in rabbits; they were only focused on the eventual preference for mirrors by observing rabbit behavior in different rearing systems. In order to assess an animal’s welfare status, it is recommended to use a combination of different welfare indicators [12]. This recommendation is based on the problems of interpretation which arise when using only a single indicator of welfare. Many of the responses that are measured as a welfare indicator could signify either a negative or positive affective state. The aim of this research was to propose a free-range farming model able to promote the physiological behavior of rabbits but also to meet the needs of the breeders, the low number of animals per pen involves the reduction of the overall available space as well as an easier control of hygienic conditions. In this context, the use of mirrors aims to detect the possible effects of a “perceived” social contact in isolated groups on rabbit behavior and stress markers levels.

## 2. Materials and Methods

### 2.1. Animals

**Figure 1:**
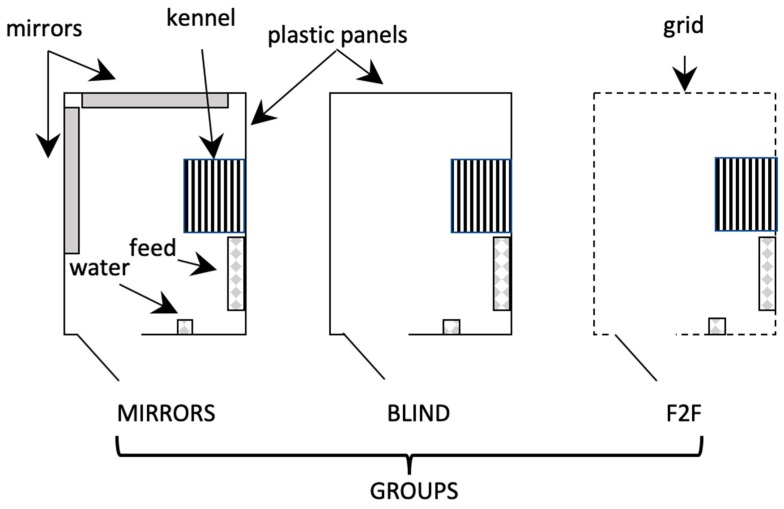
Design of the pens for mirrors, blind and face to face (F2F) groups. Each pen hosted three rabbits for nine replicates (27 rabbits for each group).

Eighty-one 45 day-old New Zealand White x California male rabbits from the same farming industry in Avellino, Italy, were included in the trial. Rabbits were clinically healthy, with a mean body weight of 1422.6 ± 15.6 g. Each rabbit was identified by using an ear tag. Animals were moved from the cages and divided into three groups (27 rabbits as nine replicates of three rabbits per group). Each group was stabled into nine pens (1.5 × 2.0 m, three for each pen) having the following features: face to face (F2F) group—the rabbits were allowed to see each other as well as the surrounding space and to have olfactory contact, blind group—they were not allowed to look around by the use of blackout plastic panels, mirrors group—they were not allowed to see either the surroundings nor the other groups but two mirrors (1.2 × 0.4 m) were placed on two adjacent walls in each box. As depicted in Figure 1, in each pen there was a kennel, a feeder area (including three troughs) and a drinking area (including three nipples). The pens were on a solid ground floor and were united for the long side of the perimeter in the order showed in Figure 1.

The panels were made of hard plastic, 0.5 cm thick, odorless and had a flat surface. Every day at 10 am the pens were cleaned, and water and feed were provided always by the same operator. A commercial feed for meat rabbits (crude protein (CP) 15.6, ether extract (EE) 3.6, crude fiber (CF) 19.0% as feed) was used all along the trial. The feed intake was recorded for the entire period of the trial by weighing the administered, refused and scattered feed. The environmental temperature and humidity were recorded along the trial.

All animals were free to move inside the pen, the duration of the trial was of 45 days (including seven days for adaptation), which is considered as the standard fattening period of rabbits reared for meat production in Italy.

After one week of adaptation, a 24 h recording was performed every seven days by using an AP-320S C&Xanadu camera for each pen. During the recording nobody had access to the pens to avoid interfering with the normal behavioral repertoire adopted by the animals. Recordings were watched by a single operator and analyzed for the following behaviors: drinking (minutes), feeding (minutes), olfactory investigation (minutes), gnawing (minutes), nose to nose (seconds), scratching (minutes), alarm (seconds), allo-grooming (minutes), kennel stay (number), stretching (seconds), locomotion (minutes), inactivity (minutes) and cecotrophy (number). For each pen, how many times and for how long the rabbits showed each of the above-mentioned behavioral repertoire was measured.

At 90 days of age, blood samples were collected fasting from an ear vein of each rabbit by the same veterinary practitioner in plastic tubes with and without K3-EDTA. K3-EDTA were used for packed cell volume (PCV) assay by using an automatic counter (HeCoVet C, SEAC, Italy). The other aliquot was centrifuged at 2000× g for 15 min and serum was used for determination of cortisol, glucose, lactate and total protein. An AIA-360 automated immunoassay analyzer and reagents from Tosoh (San Francisco, CA, USA) were used to assay cortisol. Glucose, lactate and total protein were assayed by means of an automatic biochemical analyzer AMS AUTOLAB (Rome, Italy) using reagents from Spinreact (Girona, Spain). All the procedures used in the study were approved by the Ethical Animal Care and Use Committee of the University of Naples Federico II and received formal institutional approval in accordance with local and national law, regulations and guidelines (OPBA, CSV, University of Naples Federico II, prot. 2019/0058989).

Results were expressed as means + root mean squared error (RSME) and analyzed for groups (F2F, blind, mirrors) and for daytime (time slot 1: 8:00 am–4:00 pm; time slot 2: 4:00 pm–12:00 pm; time slot 3: 12 pm–8 am).

### 2.2. Statistical Analysis

Data were analyzed by means of ANOVA (PROC GLM of SAS, 2000). Data concerning the stress markers were compared by ANOVA for one-way according to the following model:

yij = μ + Gi + εij,(1)

where: y is dependent variable, μ is the general mean, G is the effect of groups (F2F, blind, mirrors) and ε is the standard error.

The data concerning the ethogram were processed by two-way ANOVA according to the model:

yij = μ + Gi + Tj + DTij + εijk,(2)

where y is dependent variable, μ is the general mean, G is the effect of groups (F2F, blind, mirrors), T is the effect of time (time slot 1, 2, 3), DT is the first degree of interaction effect and ε is the standard error. The comparison between the means was performed using the Tukey’s test (SAS, 2000). The results were expressed as mean and the significance level was set at *p* ≤ 0.01.

## 3. Results

No stereotypic behavior was observed among rabbits. The rabbits’ ethogram is reported in Table 1.

The rabbits kept in pens F2F appeared more active compared to the other groups, they spent daily only 135.6 min to rest (*p* < 0.01) and spent much more time in gnawing (47.7 min vs. 14.8 and 13.1 min than blind and mirrors, respectively; *p* < 0.01) and olfactory investigation (30.6 min vs. 22.2 and 22.5 min of blind and mirrors, respectively; *p* < 0.01). By contrast, the mirrors and blind groups showed more similar behaviors in terms of time spent on olfactory investigation, gnawing, alarm and stretching, but always lower than F2F group. Compared to the other groups, the blind one spent more time in terms of number of kennel stay (27.6 vs. 14.7 and 2.42 for F2F and mirrors, respectively; *p* < 0.01). The mirrors group showed the higher % of time, after inactivity time and feeding, in allo-grooming (52.3 min; *p* < 0.05) compared to both the other groups. Moreover, the time spent on the mirrors was higher during the dark period (time slot 3) compared to the other time slots.

**Figure 2:**
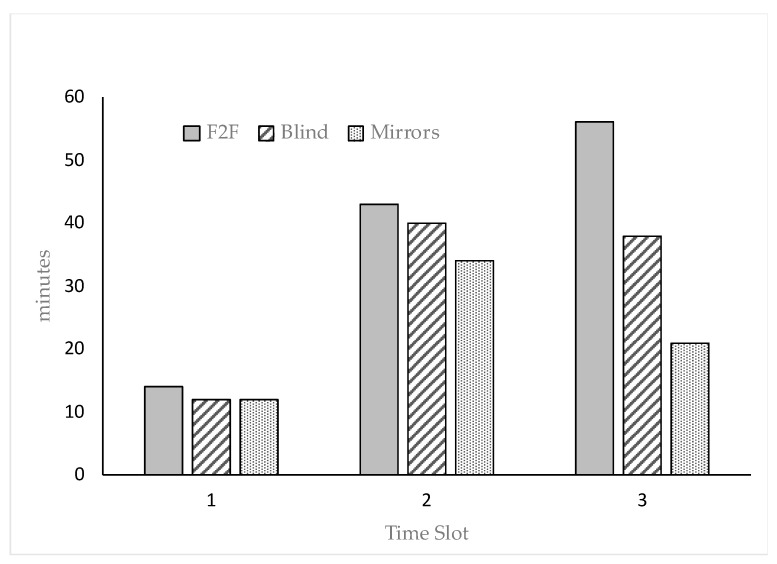
Effect of the interaction group × time of observation (time slot 1 = 8:00 am–4:00 pm; time slot 2 = 4:00 pm–12:00 pm; time slot 3 = 0:00 am–8:00 am) on the locomotory activity of the rabbits.

Despite no significant differences were detected among groups for drinking and feeding (135.6, 137.2 and 137.1 g/h/d for F2F, blind, mirrors, respectively), some differences were detected in terms of time slots. In particular, rabbits spent significantly more time drinking (64.0 min/day) from 4:00 pm to 12:00 pm (time slot 2) and less time feeding (89.5 min/day) from 8 am to 4 pm (time slot 1). In time slot 1, rabbits spent more time inactive (*p* < 0.01), in time slot 2 in olfactory investigation gnawing, nose to nose (*p* < 0.05) and less time inactive (*p* < 0.01). Interaction between group x time resulted significantly different (*p* < 0.05) only for locomotion, the differences are depicted in Figure 2. There was no significant difference among groups for the other behaviors.

In Table 2, results concerning cortisol and secondary stress markers are reported.

Glucose resulted significantly lower (*p* < 0.01) in F2F group (112.5 vs. 132.5 and 135.4 mg/dL, blind and mirrors, respectively). Total protein resulted in significantly different values (*p* < 0.01) for mirrors and blind (7.10 vs. 6.23 g/dL, respectively). Lactate resulted significantly higher (*p* < 0.05) in F2F group (81.9 vs. 65.3 and 69.2 mg/dL for F2F vs. blind and mirrors, respectively).

## 4. Discussion

The rabbits are a naturally gregarious species and wild rabbits live in small, territorial breeding groups; they are social animals [13]. The free-range breeding on the ground is a very natural form of farming, since animals are allowed to express their behavior almost completely. Despite that, most of the literature focused on extensive systems proposing different conditions, with different stocking density and groups size as well as on problems due to road transport to the slaughterhouse that may negatively affect rabbit welfare [14]. Despite its suitability to improve animal welfare, several problems that may affect production can be encountered with the free-range farming of rabbits [8]. Within them, the unfavorable hygiene conditions [15,16] and the increase of aggressiveness and hierarchies that may limit the group size [17]. Moreover, the higher locomotion activity may also negatively affect production performance [6].

In this study, all the animals proved to be not so timid thanks to the interaction with their conspecifics and to the external stimuli to which they were subjected; moreover, the rabbits seemed to be more docile and tractable during the contact with humans. As reported by Morton et al. [18], manipulating rabbits when they are bred in groups is not a problem if the animals are followed closely and receive daily attention from their managers, so they can become familiar with humans and with a part of their environment. No stereotypes were observed in any of the animals, as well as no signs of apathy. A similar proportion of activity and inactivity time in free range systems compared to wild rabbits was observed [19]. An increase in the number of activities/h of caged rabbits was found by Lehmann [20] compared to rabbits housed in semi-natural conditions and this was interpreted as restlessness. An animal is defined as ‘restless’ when it does not complete the ongoing activities, and this is a behavioral sign of increased stress in the animal. During 24 h of recording, in all groups, the greatest activity of the rabbits was observed from sunset during the night, as it is possible to see from the time marked, and this is the same behavior that occurs in nature [19]. Despite that, rabbits of blind and mirrors group were often observed inactive compared to F2F, probably because of the greater isolation of these groups from the external environment. However, it should be emphasized that none of the animals in each group showed signs of apathy.

Several differences were seen concerning rabbit behavior, comparing the three breeding systems. As reported by many authors, mirrors are able to mimic the presence of another rabbit and to reduce the stress in rabbits kept in captivity [7,10,11]. The most interesting change in terms of behavioral repertoire observed with the addition of the mirrors in the pens was the increase of allo-grooming. According to Xu [21], rabbits, being social animals, spend most of their time resting close to the others or realizing allo-grooming to their group-mates. This result is in contrast with Edgar and Seaman [11] who reported a decrease in allo-grooming in female New Zealand white rabbits bred in cages covered with mirrors. The increase seen in the present experiment supports the theory that increased levels of allo-grooming may be as a result of a lack of social stimulation. Gibb [22] reported that wild rabbits spent about 2% of their active period in allo-grooming. In our trial for all the groups we observed a percentage of time spent doing allo-grooming from 2 up to 3%, we can therefore say that for all rabbits the allo-grooming can be defined as physiological behavior and not a stereotype.

Batchelor [23] noted that wild rabbits spent very little time in the kennel to rest, whereas kennel is available to hide from predators or the group’s dominant rabbit. In our study, only the rabbits of the blind group showed a significant longer stay in the kennel but associated with a longer time also of inactivity and were, therefore, less stimulated. On the contrary, the rabbits of the other groups spent resting time in a gregarious manner and were also more active, due to the possibility of interacting with other rabbits of the neighboring enclosures, as well as for the possibility of interaction with environmental enrichment. Our results are in agreement with those of Lidfors [24], supporting the hypothesis that environmental enriching of cages or pens can reduce the frequency of gnawing and increase the grooming behavior. In addition, the time spent on the mirrors was higher during the dark period (time slot 3), in agreement with Dalle Zotte et al. [7] who found a significant (*p* < 0.001) preference of the rabbits bred in pens for mirrors during the active (dark) period.

When the animals are not in good conditions of well-being they undergo to different degrees of stress. Stress behaviors arise when the animal perceives a threat that leads it to have an abnormal, physiological and emotional state [25]. From the records we observed that the rabbits of F2F group changed behavior very frequently, they moved and assumed the alert position more than the other groups, probably due to the greater external stimuli to which they were subjected.

In order to assess the welfare state of an animal, it is advisable to use a combination of different stress indicators to avoid interpretation problems that arise when using a single observation method [12]. It is not always easy to interpret the answers as positive or negative. For this reason, it may be useful to associate the evaluation of behavioral parameters with the evaluation of markers blood parameters. Determination of blood cortisol concentration is the standard procedure to evaluate stress conditions in farm animals. Cortisol increases in blood a few minutes after the stressor and it is sustained for about one hour after the ending of the stressful event. In order to minimize the variability due to sample collection, blood was withdrawn by the same practitioner using always the same procedure.

However, the latitude of the response is not the same among the species. Cortisol and corticosterone are influenced by social stress and constitute good stress indicators in rabbits as reported by Szeto et al. [26]. No differences in cortisol levels were detected in our experiment, and results fell in the normal range for rabbits [27]. The limits of blood markers for the evaluation of stress in our rearing conditions were also confirmed by the contrasting results obtained for the secondary stress markers. Mazzone et al. [28] and Choe and Kim [29] studied a variation in glucose and lactate levels, PCV and total proteins in serum as indicators of stress in rabbits and swine, respectively. In our study, glucose was significantly lower in the F2F group, and this may due to the increased locomotion of F2F respect to the other two groups. Probably for the same reason, lactate was significantly higher in F2F group, but such result is in contrast with several authors who reported a usual linear correlation between the glucose and lactate increases [30,31]. Additionally, similarly to cortisol, no differences were seen for PCV. Packed cell volume, together with total proteins was reported to increase during stressful conditions, such as transport, because of dehydration due to several factors such as time without water, increased respiration rates and urinary water loss [31]. Overall, our results suggest that for the free-range breeding, the commonly used stress markers can be useful in evaluating metabolic responses but stress linked variations.

The behavioral patterns suggested that the use of mirrors may be effective in ameliorating rabbit welfare. Among the three groups, the F2F one showed a greater incidence, respect to the blind group, of important behaviors such as allo-grooming, OI, gnawing, alarm and scratching that reveal a more natural behavioral repertoire, thus, more respectful of rabbit behavior. Despite a similar time spent in carrying out behaviors such as: OI, gnawing, alarm and stretching of the blind group compared to mirrors one, this last group showed more time spent to allo-grooming and less in kennel stay, suggesting greater activity of the rabbits belonging to the mirrors group due to the higher stimulation caused by the mirrors in the pens. Thus, mirrors may represent a useful, economic and not invasive tool to reduce the stress of rabbits kept in captivity. The importance of establishing a kind of visual social relationship has been also underlined for the disposition of the animals in cage rows, as in the industrial systems [32]. Those authors showed a tendency of rabbits to look at each other also when located in different cages, and the preference to look towards the subject nearby was highly significant (*p* < 0.001). Also, some authors [33,34,35] observed that the individually housed rabbits were not alone because visual, olfactory and acoustic contact was still possible among the rabbits in different cages and physical contact with their kits and with the adjacent rabbit by laying against the common wall in contact with each other through the wire. These results may explain the better expression of natural behavioral repertoire in the F2F group, and, consequently, the possibility to obtain a similar goal by using mirrors.

In nature, rabbits spend most of their time with breed conservation activities (i.e., eating, drinking, reproducing and defending as attention) while in breeding conditions rabbits are mainly bored and if the gnawed sticks are not available, they gnaw the cage, the power supply or even one of the others [8]. According to literature, rabbits that spend more time in gnawing activity are more active and spend more time with investigative behavior and less time at rest [36].

Interestingly, the activity behaviors (locomotion, inactive, stretching) were higher in F2F group respect to the other two groups. Thus, the “visual” isolation from the other animals and the environment modified rabbits’ behavior, but the difference was not significant for locomotion between F2F and mirrors. This may indicate that mirrors may partially reduce the effects of isolation, but such hypothesis need to be further investigated. Importantly, such result may also have effects on energy balancing and, as a consequence, on breeding’ productive performance.

## 5. Conclusions

In conclusion, mirrors may represent a useful, low cost environmental enrichment in the free-range breeding system by acting on the behavioral repertoire with potential effects on animal welfare and productive performance.

## Figures and Tables

**Table 1:** Rabbits behavioral repertoire (means of four 24 h recordings). Time slot: 1 = 8:00 am–4:00 pm; time slot 2 = 4:00 pm–12:00 pm; time slot 3 = 0:00 am–8:00 am.

Behavioral Repertoire	Group			Time Slot			RMSE	*p*-Values		
	F2F	Blind	Mirrors	1	2	3		Group	Time	G × T
Drinking, min	43.9	45.1	40.0	31.8 ^B^	64.0 ^A^	33.3 ^B^	10.71	0.8127	0.0006	0.2264
Feeding, min	135.6	137.2	137.1	89.5 ^B^	157.9 ^A^	156.4 ^A^	20.68	0.9339	0.0003	0.1944
OI, min	30.6 ^a^	22.2 ^b^	22.5 ^b^	13.8 ^B^	37.9 ^A^	23.6 ^A,B^	6.08	0.0325	0.0039	0.7484
Gnawing, min	47.7 ^A^	14.8 ^B^	13.1 ^B^	19.9 ^b^	36.6 ^a^	19.0 ^b^	10.72	0.0004	0.0126	0.9666
N2N, s	5.50	3.25	4.08	2.42 ^b^	7.17 ^a^	3.25 ^b^	1.64	0.6190	0.0173	0.4829
Scratching, min	4.08 ^A^	2.58 ^B^	6.00 ^A^	3.00 ^B^	7.33 ^A^	2.33 ^B^	2.81	0.0028	<0.0001	0.1252
Alarm, s	20.5 ^A^	5.08 ^B^	6.75 ^B^	1.25 ^B^	18.3 ^A^	12.8 ^A^	6.61	0.0008	0.0006	0.1283
Allo-grooming, min	44.7 ^a,b^	43.0 ^b^	52.3 ^a^	29.3 ^B^	46.6 ^A,B^	64.2 ^A^	4.74	0.0407	0.0022	0.9688
Kennel stay, n	14.7 ^B^	27.6 ^A^	2.42 ^C^	15.6	10.8	18.3	8.73	0.0023	0.5126	0.1660
Stretching, s	14.0 ^a^	10.6 ^b^	11.3 ^b^	6.75 ^B^	15.3 ^A^	13.8 ^A^	0.65	0.0350	0.0071	0.6846
Locomotion, min	37.9 ^a^	25.1 ^b^	28.5 ^a,b^	13.42 ^B^	39.67 ^A^	38.42 ^A^	11.38	0.0280	<0.0001	0.0426
Inactive, min	135.6 ^B^	196.2 ^A^	173.0 ^A^	277.5 ^A^	87.0 ^C^	140.3 ^B^	9.36	0.0034	<0.0001	0.4872
Cecotrophy, n/day	6.75	10.25	10.00	-	-	-	1.84	0.0625	-	-
Mirrors, min	-	-	22.	5.75 ^b^	8.75 ^a^	8.00 ^a^	0.65	-	0.0381	-

OI: olfactory investigation; N2N: nose to nose; ^A,C^ Values within a row with different superscripts differ significantly at *p* < 0.01. ^a,b^ Values within a row with different superscripts differ significantly at *p* < 0.05. RMSE: root mean square error. G × T: group × time

**Table 2:** Cortisol and secondary stress markers.

Blood Parameters	Group			RMSE	*p*-Value
	F2F	Blind	Mirrors		
Cortisol, μg/dL	280.2	310.6	223.5	128.5	0.4040
PCV, %	37.7	41.0	40.8	4.95	0.3416
Glucose, mg/dL	112.5 ^B^	132.5 ^A^	135.4 ^A^	11.5	0.0007
Total Protein, g/dL	6.63 ^A,B^	7.10 ^A^	6.23 ^B^	0.40	0.0013
Lactate, mg/dL	81.9 ^a^	65.3 ^b^	69.2 ^b^	8.24	0.0480

PCV: packed cell volume; ^A,C^ Values within a row with different superscripts differ significantly at *p* < 0.01. ^a,b^ Values within a row with different superscripts differ significantly at *p* < 0.05. RMSE: root mean square error.
